# Effects of *Datura stramonium* L. Invasion into Different Habitats on Native Plant Functional Traits and Soil Carbon, Nitrogen and Phosphorus Stoichiometric Characteristics

**DOI:** 10.3390/biology12121497

**Published:** 2023-12-06

**Authors:** Juan Lv, Haitao Wang, Na Chang, Huaiyue Li, Cong Shi

**Affiliations:** 1 School of Environmental Science and Engineering, Tiangong University, Tianjin 300387, China; 2230040591@tiangong.edu.cn (J.L.); wanghaitao@tiangong.edu.cn (H.W.); 2130040552@tiangong.edu.cn (H.L.); 2 School of Chemical Engineering and Technology, Tiangong University, Tianjin 300387, China; changna@tiangong.edu.cn

**Keywords:** *Datura stramonium*, functional traits, invasion plants, native plants, stoichiometric characteristics of soil carbon, nitrogen and phosphorus

## Abstract

**Simple Summary:**

In this study, we ask whether the invasive *Datura stramonium* bring about effects on native plants in three different habitats, including farmland, wasteland and roadside. Studying the invaded and uninvaded areas in these habitats, we found that the invasion of *D. stramonium* brought about corresponding effects on the functional traits of native plants and the stoichiometric characteristics of soil carbon, nitrogen and phosphorus. These findings imply that invasion by *D. stramonium* has a great effect on the local ecosystem, and it has a high ability to capture resources. *D. stramonium* can improve its own competitiveness by enhancing invasiveness by changing the functional traits of native plants and the stoichiometric characteristics of soil C, N and P, which may be the reason for its invasive success.

**Abstract:**

*Datura stramonium* is an invasive herb of the family Solanaceae from Mexico and has been invading seriously in China. The effects of invasive plants on the functional traits of native plants and the stoichiometric characteristics of soil carbon, nitrogen and phosphorus in different habitats were explored by taking the invasive plant *D. stramonium* and coexisting native plants as the research object. The species, quantity and height of plants in sample plots in farmland, wasteland and roadside habitats were investigated and the specific leaf area (SLA), leaf carbon content (C), nitrogen content (N), carbon-to-nitrogen ratio (C:N), construction cost (CC_mass_) and stoichiometric characteristics of soil carbon (C), nitrogen (N) and phosphorus (P) were analyzed. The results showed that compared with the noninvaded area, the species and quantity of native plants decreased in the invaded area, and SLA and leaf N showed a decreasing trend. The plant height of native plants in the invaded area of the farmland and wasteland decreased by 23.19% and 15.26%, respectively, while the height of native plants along the roadside increased by 95.91%. The leaf C:N ratio of native plants in the invaded area along the roadside significantly increased by 54.07%. The plant height and leaf N of *D. stramonium* in the three habitats were higher than those of the native plants. The soil N in the invaded area of the three habitats increased, with the soil N in the farmland increasing by 21.05%, in the wasteland increasing by 9.82% and along the roadside significantly increasing by 46.85%. The soil carbon-to-phosphorus ratio (C:P) in the three habitats showed an increasing trend. The soil C:P ratio in the farmland increased by 62.45%, in the wasteland it increased by 11.91% and along the roadside it significantly increased by 71.14%. These results showed that invasion by *D. stramonium* has a great effect on the local ecosystem, and it has a high ability to capture resources. *D. stramonium* can improve its own competitiveness by enhancing invasiveness by changing the functional traits of native plants and the stoichiometric characteristics of soil C, N and P, which may be the reason for its invasive success.

## 1. Introduction

Biological invasion is one of the important parts of global change. The spread of invasive species seriously threatens the biodiversity, structure and function of the ecosystem in invaded areas [1,2] and brings enormous losses to society and the economy [3,4]. At present, invasive species have become the second major cause of biodiversity loss after habitat destruction [5,6]. China has a vast territory, with considerable differences in physiographic conditions in different regions. Most exotic plants from all over the world can find a suitable growth environment within the Chinese territory and thus cause invasion [7]. In recent years, with the increase in human activities, the problem of biological invasion has become increasingly serious. According to statistics, among the alien invasive species invading China’s agriculture, forestry and water ecosystems, terrestrial plants account for approximately 44% and aquatic plants account for approximately 7% and have had serious effects on the biodiversity, ecological environment, economy and society in China [8].

Plant functional traits refer to the plant traits that can respond to environmental changes and affect ecosystem functions [9]. The arrival of alien plants disrupts the original balance of the ecosystem, directly affects native species through the formation of new interspecific relationships (e.g., competition, predation and parasitism) and indirectly affects native species through the disturbance and destruction of resources [10]. Native species also develop new adaptive features in response to the arrival of alien species [11]. The soil constitution is frequently thought to be connected to plant invasion [12]. Carbon and nitrogen play an important role in the growth of plants. Carbon is the main nutrient for building the skeleton and structure of plants, while nitrogen is the main component of protein, which is crucial for plant growth and reproduction. Carbon and nitrogen, as limiting nutrients of plant growth, restrict each other. By measuring the carbon and nitrogen content of plants, their resource utilization strategies can be quantified and their ecological strategies better explained. With the deeper understanding of the importance of underground processes in ecosystems, an increasing number of researchers have begun to pay attention to the effects of alien plant invasion on soil physical and chemical properties [13,14]. The effects of alien plants on the structure of the local plant community will inevitably lead to changes in soil physical and chemical properties, which may be conducive to alien plants acquiring advantage in competition with native plants, thus intensifying the harm of invasion by alien plants [15]. Therefore, understanding the effects of alien plants’ invasions on the stoichiometric characteristics of soil is of great significance for revealing the invasion mechanism, effectively managing invasive plants and restoring and rebuilding damaged ecosystems.

*Datura Stramonium* is originally from Mexico. It is an annual upright herb of the family Solanaceae that is widely distributed in temperate and tropical regions. The whole *D. stramonium* plant contains alkaloids, which can cause poisoning when ingested by humans and livestock, especially the fruit and seeds. *D. stramonium* is larger in size and has a strong growth capacity; in particular, it has a considerable allelopathic inhibition effect on the growth of coexisting plants [16]. As a malignant and harmful weed, *D. stramonium* mainly invades farmland and wasteland, has strong environmental adaptability and potential for invasion and is resistant to drought, soil infertility and pollution [17]. *D. stramonium* is an invasive species whose range has expanded greatly in recent years in Romania [18], and studies have shown that, in 2024, the invaded area of *D. stramonium* may reach 130100 square kilometers in Liaoning Province, China [19]. In addition to harming the biodiversity of the local ecosystem, it can also spread diseases and pests and pose a threat to the agricultural production, natural ecosystem and human living environment of the invasion site. At present, studies on *D. stramonium* have mainly focused on its accumulation of heavy metals, allelopathy and chemical compounds, but the species has only been mentioned in a very small number of regional invasive plant investigations [20,21]. Therefore, our study uses the invasive plant *D. stramonium*, native plants and soil as the research object, investigates the species, quantity and height of plants in a noninvaded area and invaded area of farmland, wasteland and roadside habitats and analyzes the specific leaf area (SLA), leaf carbon content (C), nitrogen content (N), carbon-to-nitrogen ratio (C:N), leaf construction cost (CCmass) and the stoichiometric characteristics of soil carbon (C), nitrogen (N) and phosphorus (P) to determine the effects of *D. stramonium* invasion on native plants and soil stoichiometric characteristics in different habitats to elucidate the invasive mechanism of *D. stramonium* and provide a scientific basis for ecological prevention and control.

## 2. Materials and Methods

### 2.1. Study Area

The study area is located in Wuqing District, Tianjin, China (117°18′ E, 39°22′ N), and has a warm temperate subhumid continental monsoon climate. The average annual temperature is 11.6 °C, the average temperature in January is −5.1 °C, the average temperature in July is 26.1 °C, the average annual precipitation is 606 mm, the frost-free period is 212 days and the annual sunshine duration is 2752 h. *D. stramonium* is an annual herb, and the annual new seedlings are reproduced by seeds. The experimental areas are three habitats invaded by *D. stramonium*, including farmland (FL), wasteland (WL) and roadside (RS). *D. stramonium* mainly invades farmland and wasteland and has a strong environmental adaptability and potential for invasion. There are more disturbances at the roadside compared to farmland and wasteland. The three habitats are more representative. In order to ensure that the elevation, precipitation and temperature of the three habitats are similar, the distance between the study areas of different habitats is not more than 10km. No prevention measures have been carried out in the experimental area in the past five years, and there is no human interference. *D. stramonium* has heavily invaded each study area; that is, *D. stramonium* was the dominant population in the community, and its cover was more than 60%. Each habitat was divided into two sample plots according to whether there was an invasion of *D. stramonium*: (1) Noninvaded area, that is, there is no *D. stramonium* in the sample plot and only native plants grow. (2) Invaded area, that is, there are *D. stramonium* and native plants growing in the sample plot, and the *D. stramonium* covers was more than 60%. The interval between the noninvaded and invaded area under the same habitat did not exceed 50 m to ensure the basic consistency of other conditions except vegetation factors.

### 2.2. Sample Collection and Measurement

In each sample plot, three quadrats (10 m × 10 m) were randomly set up, with intervals of 10–15 m between each quadrat. Five subquadrats (1 m × 1 m) were randomly set up in each quadrat, the quantity and cover of all species (including the invasive plant *D. stramonium* and native coexisting plants) in each subquadrat were recorded, and the heights of all plants were measured. In each subquadrat, a number of well-growing *D. stramonium* and native plants were selected, and 10–20 mature and healthy leaves were randomly collected from these plants. The leaves of the same plant were mixed into one sample, which was packed in an envelope and taken to the laboratory. The samples were kept for 15 min at 105 °C and dried to a constant weight at 55 °C for the measurement of functional traits. After the plants and litter on the surface of the soil were removed, soil samples were collected by using a 5-point sampling method in each subquadrat, placed into self-sealing plastic bags and taken to the laboratory. After being air-dried and ground, the samples were screened, sealed and stored at room temperature for the measurement of soil carbon, nitrogen and phosphorus content. The leaf area was measured by a YMJ-B leaf-area-measuring instrument. The total carbon and nitrogen contents in leaves and soil were measured by a Vario MICRO Cube element analyzer (Elementar, Germany). The total phosphorus content in the soil was measured by molybdenum–antimony colorimetry.

### 2.3. Statistical Analysis

SPSS Statistics statistical software, version 25.0.0 (IBM SPSS Statistics, IBM, SPSS Inc., Armonk, NY, USA), was used to analyze the statistics data. The effects of the invasive plant on the stoichiometric characteristics of soil carbon, nitrogen and phosphorus were estimated using a one-way and two-way analysis of variance (ANOVA). The significance level was set at *p* < 0.05.

## 3. Results

### 3.1. Community Composition in Different Habitats

The native plants in different habitats were all common plants in the study area. A total of 28 species of native coexisting plants from 15 families were found in the survey (Table 1). Among them, Poaceae and Compositae were the most abundant, with 7 and 6 species, respectively. The other families were Chenopodiaceae, Malvaceae, Leguminosae, Asclepiadaceae, Moraceae, Portulacaceae, Polygonaceae, Euphorbiaceae, Amaranthaceae, Lamiaceae, Equisetaceae, Rubiaceae and Zygophyllaceae. These families have only one or two species of plants in the study area (Table 1). There are 28 species of plants along the roadside; the farmland habitat was the second most species-rich, with 24 species of plants; and the wasteland habitat had the least species, with 16 (Table 1). Compared with the noninvaded area, the species and quantity of native plants in the invaded area decreased, 10 species decreased along the roadside, 6 species decreased in the farmland and 4 species decreased in the wasteland (Table 1).

### 3.2. Effect of D. stramonium Invasion into Different Habitats on the Functional Traits of Native Plants

**Figure 1:**
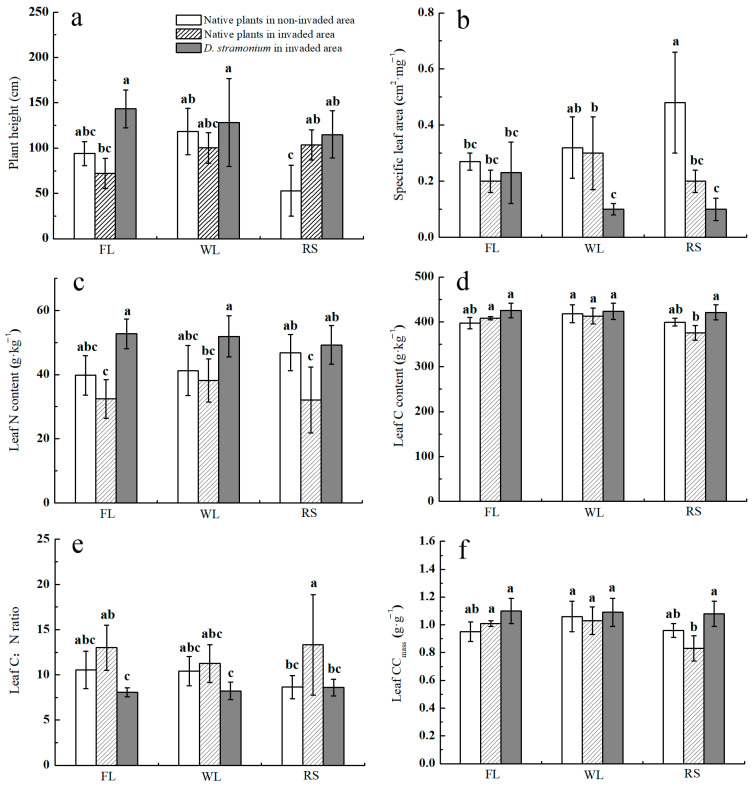
Effects of invasion of *D. stramonium* in different habitats on functional traits of native plants. (**a**): Plant height; (**b**): specific leaf area (SLA); (**c**): leaf N content; (**d**): leaf C content; (**e**): leaf C:N ratio; (**f**): leaf construction cost (CC_mass_). Different lowercase letters indicate significant difference treatments for each habitat (*p* < 0.05).

In the noninvaded area, the plant height of native plants in the farmland and wasteland was significantly higher than that along the roadside (Figure 1a). Compared with that in the noninvaded area, the plant height of native plants in the invaded area of the farmland and wasteland decreased by 23.19% and 15.26%, respectively, while the height of native plants along the roadside increased by 95.91% (Figure 1a). The plant height of *D. stramonium* in both the noninvaded area and invaded area of the three habitats was higher than that of native plants (Figure 1a). In the noninvaded area, the native plants along the roadside had the largest SLA (Figure 1b). Compared with that in the noninvaded area, the SLA of native plants in the invaded area of the three habitats showed a decreasing trend, among which the SLA along the roadside decreased significantly by 55.17% (Figure 1b). In the wasteland and roadside, the SLA of *D. stramonium* in the invaded area was significantly lower than that of native plants in both the noninvaded and invaded area (Figure 1b). In the noninvaded area, there was no significant difference in leaf N among native plants in the three habitats (Figure 1c). Compared with the noninvaded area, the leaf N of native plants in the invaded area of the three habitats showed a decreasing trend, among which the leaf N of native plants along the roadside significantly decreased by 31.49% (Figure 1c). The leaf N of *D. stramonium* in the three habitats was higher than that of the native plants in both the noninvaded and invaded area (Figure 1c). Along the roadside, the leaf C and CCmass of *D. stramonium* in the invaded area were significantly higher than those of native plants (Figure 1d), and the other two habitats also showed an increasing trend, but the difference was not significant (Figure 1d,f). In the noninvaded area, the leaf C:N ratio of native plants along the roadside was relatively low (Figure 1e). Compared with the noninvaded area, the leaf C:N ratio of native plants in the invaded area along the roadside significantly increased by 54.07%, and others were also on the rise. (Figure 1e). The leaf C:N ratio of *D. stramonium* was significantly lower than that of native plants in the invaded area (Figure 1e).

### 3.3. Effect of D. stramonium Invasion into Different Habitats on the Stoichiometric Characteristics of Soil Carbon, Nitrogen and Phosphorus

**Figure 2:**
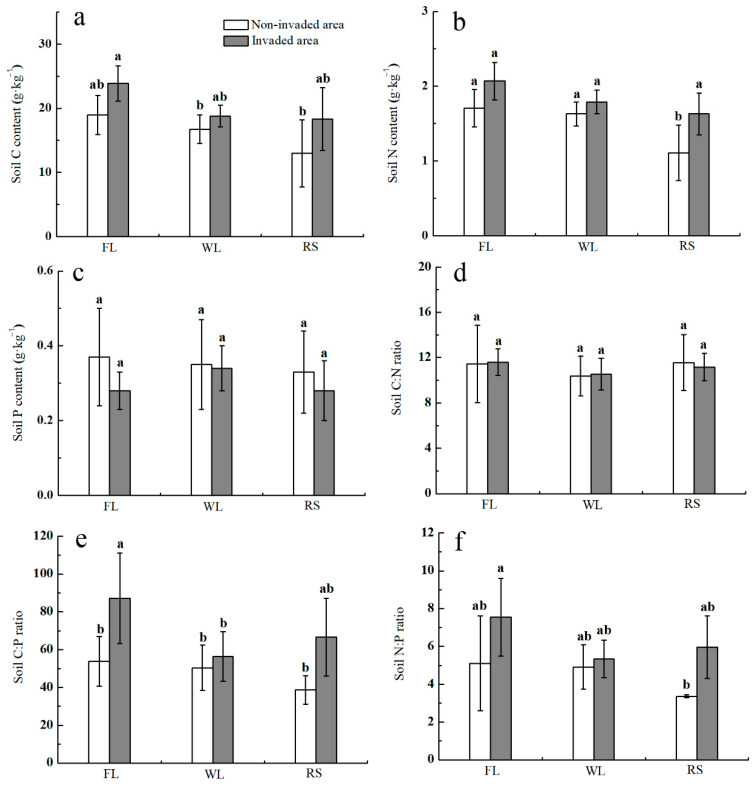
Effects of invasion of *D. stramonium* in different habitats on stoichiometric characteristics of soil carbon, nitrogen and phosphorus. (**a**): Soil C content; (**b**): soil N content; (**c**): soil P content; (**d**): soil C:N ratio; (**e**): soil C:P ratio; (**f**): soil N:P ratio. Different lowercase letters indicate significant difference treatments for each habitat (*p* < 0.05).

In the noninvaded area, the soil N content in the three habitats was as follows: farmland > wasteland > roadside (Figure 2b). Compared with the noninvaded area, the soil N in the invaded area of the three habitats increased, with the soil N in the farmland increasing by 21.05%, in the wasteland increasing by 9.82% and along the roadside significantly increasing by 46.85% (Figure 2b). In the noninvaded area, the soil C content in the three habitats was ranked as farmland > wasteland > roadside, but the difference was not significant (Figure 2a). Compared with the noninvaded area, the soil C in the invaded area of the three habitats showed an increasing trend: the soil C in the farmland increased by 25.87%, in the wasteland it increased by 12.24% and along the roadside it increased the most, by 41.18%, but there was no significant difference (Figure 2a). In the noninvaded area, there was little difference in the soil P under different habitats (Figure 2c). Compared with the noninvaded area, the soil P in the invaded area of all habitats decreased, with the soil P in the farmland decreasing by 24.32%, in the wasteland decreasing by 1.78% and along the roadside decreasing by 13.90%, but there was no significant difference (Figure 2c). In the noninvaded area, the soil N:P ratio along the roadside was relatively low (Figure 2f). Compared with the noninvaded area, the soil N:P ratio increased in the invaded area of all habitats, with an increase of 47.83% in the farmland, 8.53% in the wasteland and 77.80% along the roadside, but there was no significant difference (Figure 2f). There was no significant difference in the soil C:N ratio in the three habitats (Figure 2d). In noninvaded area, the soil C:P ratio along the roadside was relatively low (Figure 2e). Compared with the noninvaded area, the soil C:P ratio in the invaded area of the three habitats showed an increasing trend. The soil C:P ratio in the farmland significantly increased by 62.45%, in the wasteland it increased by 11.91% and along the roadside it increased by 71.14% (Figure 2e). The soil C and N were significantly affected by habitat type, and the soil C, N, N:P ratio and C:P ratio were significantly affected by *D. stramonium* invasion (Table 2).

### 3.4. Relationship between Functional Plant Traits and Soil Carbon, Nitrogen and Phosphorus Stoichiometric Characteristics

The relationship between the functional traits of plants and the stoichiometric characteristics of soil carbon, nitrogen and phosphorus varied with the invasion of *D. stramonium* into different habitats. The soil C, P and C:N ratio had a significant effect on functional traits (Table 3, Table 4 and Table 5). In the invaded area of farmland, the soil C was significantly positively correlated with the plant height and leaf N, and significantly negatively correlated with the leaf C:N ratio, the soil P was significantly negatively correlated with SLA and the soil C:N ratio was significantly positively correlated with SLA (Table 3). In the noninvaded area of wasteland, the soil C was significantly positively correlated with the leaf C and CCmass, while the soil P was significantly positively correlated with the leaf C:N ratio (Table 4). In the noninvaded area of farmland, the soil C:N ratio was significantly positively correlated with the plant height and leaf C:N ratio (Table 3). Conversely, functional traits were regulated by different soil characteristics in different habitats. In the noninvaded area of farmland, the leaf C:N ratio and soil C:N ratio showed a significant positive correlation (Table 3). In the invaded area of farmland, the leaf C:N ratio was significantly negatively correlated with the soil C (Table 3). In the noninvaded area of wasteland, the leaf C:N ratio was significantly positively correlated with the soil P (Table 4). Compared with the noninvaded area, the relationship between functional traits and soil characteristics differed in the invaded area of the three habitats: it was closer in the invaded area of farmland (Table 3), weaker in the invaded area of wasteland, and there was no difference along the roadside (Table 5). 

## 4. Discussion

Many studies have shown that invasive plants can significantly change the structure and function of local ecosystems. For example, the invasion of *Solidago canadensis* resulted in more than 30 types of native species (accounting for 1/10 of the native species in Shanghai) disappearing locally in Shanghai, China [22]. After *Spartina alterniflora* invaded coastal beaches in Fujian, China, and other places, mangrove wetland ecosystems were severely damaged. Fish, shrimp, crabs, shellfish and other organisms could not live on the beaches, and more than 200 species [23] of original organisms were reduced to close to 20 species. After *Centaurea iberica* invaded Kashmir Himalaya, the species diversity was severely reduced and the homogenization of diverse plant communities appeared [24]. Our study showed that compared with noninvaded areas, the species and quantity of the local plant community decreased significantly in invaded areas. This indicates that after invasion by *D. stramonium*, the competitive advantage of this plant as a dominant species became more considerable, leading to the species and quantity of local plants in the invaded area decreasing: 10 species decreased along the roadside, 6 species decreased in the farmland and 4 species decreased in the wasteland, which had a great effect on the composition of the local community and the ecosystem. In addition, compared with other habitats, plant communities along roadsides have the most species, which may be due to frequent human activities and strong interference, as well as developed transportation pathways facilitating the diffusion of plant seeds or other types of propagators into the roadside habitat [25,26].

The functional traits of plants can indicate the effect of ecosystems on environmental change, individually or collectively, and they can have a strong effect on ecosystem processes [27]. The functional traits reflect adaptations to abiotic and biotic factors and, thus, can be used to describe and predict species responses to changes in these factors [28]. Studies have shown that the comprehensive effect of functional traits and environmental factors determines the success of plant invasion [29,30]. The results of this study showed that there were significant differences in the functional traits of *D. stramonium* and native plants in different habitats. In this study, in the three habitats, the plant height of *D. stramonium* was higher than of native plants. Plant height is a major factor in the invasion success of plants, and a higher plant height allows plants to exploit more resources. A taller height makes it easier for a plant to occupy the dominant position and capture the most critical factors in the environment, such as light, which has a direct effect on the growth, development and survival of plants [31]. 

This study found that in the wasteland and on the roadside, the SLA of *D. stramonium* was significantly lower than that of native plants. Along the roadside, the leaf CCmass of *D. stramonium* was significantly higher than that of native plants, and the leaf CCmass of *D. stramonium* in the other two habitats also tended to be higher than that of native plants, but the difference was not significant. However, current studies on SLA and leaf CCmass have yielded contrasting results [32,33]. The reason may be that most studies involve only a few species, making it more likely that the results will be inconsistent due to species-specific factors. In addition, plants that can coexist with *D. stramonium* are also competitive, and the competition ability is manifested by certain functional traits (such as SLA and leaf CCmass). Such plants may appear to compete with *D. stramonium*, but this does not affect the competition ability of *D. stramonium* since it still has considerable advantages in other important functional traits.

The results showed that the leaf N of *D. stramonium* in the three habitats was higher than that of native plants. Compared with the noninvaded area, the leaf N of native plants in the invaded area of the three habitats showed a decreasing trend. When the leaf N is higher, it generally represents a strong ability to capture resources [34,35]. In addition, the higher leaf N of invasive plants may be due to their innate advantages. Studies have shown that the plant innate advantage can also promote invasion [36,37]. The results of this study showed that the leaf C:N ratio of *D. stramonium* was significantly lower than that of native plants in the invaded area. Compared with the noninvaded area, the leaf C:N ratio of native plants in the invaded area along the roadside significantly increased, and that in the other two habitats also showed an increasing trend. The difference of the leaf C:N ratio between alien invasive plants and native plants is one of the reasons why alien invasive plants have a stronger competitiveness than native plants. Alien invasive plants experience reduced selection from natural enemies (especially obligate natural enemies) and show a reduced defense investment and a strong ability to capture and utilize resources, thus resulting in a lower leaf C:N ratio.

Plant height and the leaf C:N ratio are the representative functional traits of the balance between resource allocation and utilization. In the noninvaded area of farmland, both plant height and the leaf C:N ratio were significantly positively correlated with the soil C:N ratio, further indicating the sensitivity of the dependence of functional traits on soil characteristics. In this study, these two functional traits were also regulated by different soil characteristics in different habitats. In the noninvaded farmland area, plant height and the leaf C:N ratio were significantly positively correlated with the soil C:N ratio, plant height was significantly positively correlated with the soil C:P ratio and the leaf C:N ratio was significantly negatively correlated with the soil C. In the noninvaded wasteland area, the leaf C:N ratio was significantly positively correlated with the soil P.

According to the research results for different habitats, the functional traits of plants along the roadside changed the most after the introduction of *D. stramonium*. A large number of studies have shown that the distribution of invasive plants is closely related to traffic [38,39,40]. The road system allows invasive plants to spread more easily [41,42], and the construction and maintenance of the road system not only destroys the local microecology but also provides a blank ecological niche for invasive plants. In addition, the road system also causes changes in the nearby microclimate and microenvironment, which causes stress to some native species and makes it easier for invasive plants to settle [43]. Along the road traffic, stormwater runoff and bare ground are advantageous to the invasion of invasive plants [44].

Soil is the medium through which alien invasive plants interact with native plants, and changes in soil characteristics can affect the structure and function of the ecosystem [45]. Soil nutrients are one of the key factors limiting the growth of plants, and the soil nutrient content in the microenvironment is usually closely related to the competition and succession among plants. Soil N pools and cycling change markedly when conifers invade mountain meadows [46]. In this study, it was found that the soil nutrient content, especially the soil C, P and C:N ratio, had a prominent effect on some plant functional traits. Liao et al. [47] analyzed the published literature and found that the carbon and nitrogen pool of soil in the invaded area were 7% and 19% higher than those of soil under local plants, respectively. This study also found that the soil C and N increased in the invaded area of the three habitats. Studies have shown that the spatial distribution of soil C and N is consistent, so soil C and N show the same change pattern under different environmental changes [48], which is also supported by the conclusion of this study. However, the same invasive plant may have different effects on soil characteristics in different habitats [49]. Compared with the noninvaded area, the soil C and N in the invaded area along the roadside increased in a larger proportion than those in the other two habitats. The soil P in the invaded area of all habitats decreased, while the soil N:P and C:P ratio increased, which may have been caused by the greater consumption of phosphorus in the growing season of *D. stramonium*. The study showed that when *Carpobrotus edulis* L. invades a dune ecosystem, it causes significant changes to the soil nutrient content, and the invasive plant-soil interactions also significantly affected the germination and emergence of different plant species [13]. Invasion by *D. stramonium* accelerates the soil material cycle, creates a more favorable soil environment for its own growth and competition and intensifies its invasion intensity, which may be one of the reasons for the successful invasion and rapid expansion of *D. stramonium*.

## 5. Conclusions

In this study, the changes in the functional traits of invasive and native plants, as well as the stoichiometric characteristics, were researched, and then the effects of the invasive plant *D. stramonium* on ecosystems were analyzed in different habitats. Our results indicate that the invasive plant *D. stramonium* affected the species turnover of the native plant communities, broke the biodiversity of the native plant communities and changed the trade-off strategies of the native plants in terms of resource investment and utilization, and the native plants invested more resources in defense investment. The number of native plant species along roadsides was in the greatest decline and the functional traits underwent the greatest change that represented the more invasive nature of the disturbance-prone habitat. The higher plant height, the leaf N and the lower leaf C:N ratio of *D. stramonium* represented a stronger ability to capture resources, and the invasion of *D. stramonium* had an accumulative effect on the soil N, which resulted in a more pronounced positive feedback mechanism between the plant and the soil, and an accelerated chemical cycling process of soil carbon, nitrogen and phosphorus, which resulted in a greater ability to absorb and utilize soil N compared to that of the native plants. All these results indicate that *D. stramonium* has a strong competitive ability, which enables *D. stramonium* to successfully invade, stabilize the establishment and rapidly expand. These findings will be helpful to better understand the invasion mechanisms of *D. stramonium.*

## Figures and Tables

**Table 1:** Species and quantity of native plants in different habitats.

Habitat Type	Noninvaded Area			Invaded Area		
	Species Number	Species	Quantity	Species Number	Species	Quantity
Farmland	15	*Xanthium sibiricum*	8	9	*Cirsium japonicum*	3
		*Cirsium japonicum*	2		*Phragmites australis*	17
		*Sonchus arvensis*	2		*Chenopodium album*	7
		*Artemisia capillaris*	12		*Abutilon theophrasti*	3
		*Setaria viridis*	40		*Cynanchum chinense*	2
		*Echinochloa crusgalli*	58		*Metaplexis japonica*	3
		*Digitaria sanguinalis*	5		*Humulus scandens*	18
		*Phragmites australis*	55		*Polygonum orientale*	22
		*Chenopodium album*	5		*Acalypha australis*	8
		*Kochia scoparia*	2			
		*Glycine soja*	3			
		*Cynanchum chinense*	2			
		*Metaplexis japonica*	3			
		*Humulus scandens*	8			
		*Salsola collina*	5			
Wasteland	11	*Sonchus arvensis*	2	5	*Xanthium sibiricum*	8
		*Setaria viridis*	35		*Setaria viridis*	20
		*Echinochloa caudata*	16		*Chenopodium album*	12
		*Digitaria sanguinalis*	8		*Abutilon theophrasti*	3
		*Phragmites australis*	5		*Polygonum orientale*	2
		*Chenopodium album*	10			
		*Kochia scoparia*	2			
		*Abutilon theophrasti*	3			
		*Humulus scandens*	7			
		*Polygonum orientale*	35			
		*Acalypha australis*	17			
Roadside	19	*Xanthium sibiricum*	8	9	*Xanthium sibiricum*	5
		*Sonchus arvensis*	2		*Cirsium japonicum*	12
		*Ixeris polycephala*	7		*Bidens tripartita*	7
		*Bidens tripartita*	30		*Setaria viridis*	25
		*Inula japonica*	2		*Chloris virgate*	33
		*Setaria viridis*	5		*Chenopodium album*	3
		*Chloris virgate*	17		*Kochia scoparia*	2
		*Digitaria sanguinalis*	7		*Humulus scandens*	13
		*Chenopodium album*	2		*Tribulus terrestris*	8
		*Kochia scoparia*	10			
		*Abutilon theophrasti*	4			
		*Cynanchum chinense*	5			
		*Metaplexis japonica*	2			
		*Humulus scandens*	5			
		*Portulaca oleracea*	7			
		*Acalypha australis*	2			
		*Mentha canadensis*	5			
		*Equisetum ramosissimum*	2			
		*Rubia cordifolia*	1			

**Table 2:** The *F* values for the two-way ANOVA on the stoichiometric characteristics of soil carbon, nitrogen and phosphorus.

Index	Habitat Type	Presence of Invasion	Habitat Type × Presence of Invasion
Soil C	4.0071 *	5.9178 *	0.3760
Soil N	6.4315 *	8.3023 *	0.7070
Soil P	0.2195	1.0139	0.3064
Soil C:N ratio	0.4634	0.0006	0.0393
Soil C:P ratio	2.3730	8.8378 *	1.2339
Soil N:P ratio	1.6918	5.7186 *	0.8515

Note: * show the significant difference at *p* = 0.05.

**Table 3:** The relationship between functional traits and the stoichiometric characteristics of soil carbon, nitrogen and phosphorus in the farmland.

Type	Index	Soil C	Soil N	Soil P	Soil C:N Ratio	Soil C:P Ratio	Soil N:P Ratio
Noninvaded area	Height	−0.14	−0.41	−0.72	0.84 *	0.53	0.15
	SLA	0.01	0.2	0.57	−0.59	−0.46	−0.24
	Leaf N	0.3	0.54	0.75	−0.75	−0.36	0.04
	Leaf C	−0.41	−0.64	−0.67	0.7	0.23	−0.21
	Leaf CCmass	−0.39	−0.62	−0.65	0.71	0.24	−0.21
	Leaf C:N ratio	−0.26	−0.5	−0.67	0.77 *	0.36	−0.06
Invaded area	Height	0.83 *	−0.5	−0.48	0.59	0.82 *	−0.28
	SLA	0.43	−0.80 *	−0.79 *	0.77 *	0.72	−0.46
	Leaf N	0.90 **	−0.02	0.06	0.21	0.59	−0.07
	Leaf C	0.61	−0.32	−0.27	0.4	0.57	−0.21
	Leaf CCmass	0.61	−0.32	−0.27	0.4	0.57	−0.21
	Leaf C:N ratio	−0.90 **	0	−0.12	−0.2	−0.56	0.09

Note: ** and * show the significant difference at *p* = 0.01 and *p* = 0.05.

**Table 4:** The relationship between functional traits and the stoichiometric characteristics of soil carbon, nitrogen and phosphorus in the wasteland.

Type	Index	Soil C	Soil N	Soil P	Soil C:N Ratio	Soil C:P Ratio	Soil N:P Ratio
Noninvaded area	Height	−0.32	0.43	0.07	−0.57	−0.39	0.33
	SLA	0.14	0.09	−0.29	0.19	0	0.15
	Leaf N	0.63	0.5	−0.57	0.25	0.64	0.91 **
	Leaf C	0.80 *	0.73	0.17	0.38	0.55	0.57
	Leaf CCmass	0.80 *	0.73	0.18	0.37	0.54	0.56
	Leaf C:N ratio	−0.39	−0.15	0.80 *	−0.2	−0.52	−0.75
Invaded area	Height	0.19	−0.11	−0.23	0.41	0.21	0.07
	SLA	0.59	0.37	−0.38	0.21	0.53	0.48
	Leaf N	0.09	0.58	−0.07	−0.58	0.04	0.33
	Leaf C	0.04	0.29	−0.38	−0.26	0.21	0.38
	Leaf CCmass	0.04	0.29	−0.38	−0.26	0.21	0.38
	Leaf C:N ratio	−0.19	−0.64	−0.1	0.56	0	−0.26

Note: ** and * show the significant difference at *p* = 0.01 and *p* = 0.05.

**Table 5:** The relationship between functional traits and the stoichiometric characteristics of soil carbon, nitrogen and phosphorus along the roadside.

Type	Index	Soil C	Soil N	Soil P	Soil C:N Ratio	Soil C:P Ratio	Soil N:P Ratio
Noninvaded area	Height	−0.41	−0.62	−0.58	0.49	0.05	−0.62
	SLA	0.59	0.75	0.67	−0.5	0.08	0.72
	Leaf N	0.01	0.24	0.14	−0.53	−0.33	0.29
	Leaf C	0.17	−0.19	−0.25	0.54	0.56	−0.15
	Leaf CCmass	0.16	−0.2	−0.26	0.55	0.55	−0.16
	Leaf C:N ratio	0	−0.32	−0.24	0.68	0.47	−0.37
Invaded area	Height	0.28	0.09	−0.51	0.19	0.43	0.29
	SLA	−0.23	−0.41	−0.4	0.49	−0.1	−0.29
	Leaf N	0.23	0.35	0.23	−0.54	0.16	0.33
	Leaf C	0.19	0.19	0.16	−0.02	0.13	0.15
	Leaf CCmass	0.19	0.19	0.16	−0.02	0.13	0.15
	Leaf C:N ratio	−0.27	−0.45	−0.32	0.7	−0.18	−0.41

## Data Availability

The data presented in this study are available on request from the corresponding author.
